# Torsional behavior of chromatin is modulated by rotational phasing of nucleosomes

**DOI:** 10.1093/nar/gku694

**Published:** 2014-08-06

**Authors:** Gi-Moon Nam, Gaurav Arya

**Affiliations:** Department of NanoEngineering, University of California, San Diego, 9500 Gilman Drive, La Jolla, CA 92093-0448, USA

## Abstract

Torsionally stressed DNA plays a critical role in genome organization and regulation. While the effects of torsional stresses on naked DNA have been well studied, little is known about how these stresses propagate within chromatin and affect its organization. Here we investigate the torsional behavior of nucleosome arrays by means of Brownian dynamics simulations of a coarse-grained model of chromatin. Our simulations reveal a strong dependence of the torsional response on the rotational phase angle *Ψ*_0_ between adjacent nucleosomes. Extreme values of *Ψ*_0_ lead to asymmetric, bell-shaped extension-rotation profiles with sharp maxima shifted toward positive or negative rotations, depending on the sign of *Ψ*_0_, and to fast, irregular propagation of DNA twist. In contrast, moderate *Ψ*_0_ yield more symmetric profiles with broad maxima and slow, uniform propagation of twist. The observed behavior is shown to arise from an interplay between nucleosomal transitions into states with crossed and open linker DNAs and global supercoiling of arrays into left- and right-handed coils, where *Ψ*_0_ serves to modulate the energy landscape of nucleosomal states. Our results also explain the torsional resilience of chromatin, reconcile differences between experimentally measured extension-rotation profiles, and suggest a role of torsional stresses in regulating chromatin assembly and organization.

## INTRODUCTION

DNA is constantly subjected to twisting forces *in vivo*, such as those generated by DNA unzipping and rezipping during transcription and replication (1,2). Until recently, torsional stresses and the resultant supercoiling of DNA were viewed as a byproduct of biological processes that needed to be eliminated through specialized enzymes called topoisomerases. However, emerging evidence suggests that torsional stresses might serve important roles in gene regulation by: melting twist-sensitive DNA sequences to promote or suppress transcription factor binding (2,3); facilitating the formation of higher-order structures like coils, solenoids and plectonemes for mediating long-range interactions (4) and maintaining global gene expression patterns (5); and promoting the assembly (6,7), disassembly (8–10) and remodeling of nucleosomes (9,11). While the effects of torsional stresses on naked DNA have been extensively studied (12–19), DNA is rarely present in its naked form in eukaryotic organisms. Instead, it is organized into chromatin (20), a fiber made up of alternating units of ∼146 bp-long stretches of DNA wrapped around histones, yielding nucleosomes and ∼20–80 bp-long stretches of naked DNA known as linker DNAs.

So far, only a handful of studies have examined the torsional behavior of chromatin (9,21–23). These studies, measuring the extension of *in vitro*-reconstituted nucleosome arrays as a function of external twisting (rotation) of the array ends, revealed several unique features of nucleosome arrays that are markedly different from those of naked DNA. Most notably, the arrays were significantly more torsionally resilient than DNA, capable of accommodating large amounts of imposed rotations without significant changes in array extension (21). Moreover, the measured extension-rotation curves exhibited asymmetric shapes, with the maximal array extension shifted toward negative (9,21,23) or positive rotations (22). The shape and negative shift of the extension-rotation curves was explained in terms of a statistical model (21) in which nucleosomes exhibited three discrete states with open, negatively-crossed and positively-crossed linker DNAs (24,25). Each state was assigned a distinct free energy and linking number, and contributed a different length to array extension. The observed changes in array extension with imposed rotations were then presumed to occur due to rotation-induced shifts in the population of nucleosomes residing in each state. Nucleosomal transitions between the three states were also used to explain why arrays might be able to absorb external rotations with small change in extension.

While the above studies have provided the first insights into the torsional behavior of nucleosome arrays, the detailed internal structure and dynamics of negatively and positively supercoiled arrays remains elusive. What is the conformation of linker DNAs, array handedness and nucleosome arrangement in supercoiled arrays? How do torsional stresses propagate along the array through local and global structural changes? How does the torsional response of the arrays depend on their intrinsic topology? These questions are challenging to address experimentally, due to difficulties in simultaneously imaging and torsionally manipulating nucleosome arrays, and understandably so, only global features of supercoiled arrays such as their extension have been probed so far. The questions also fall beyond the scope of the model proposed so far (21), which presents a highly simplified, statistical view of supercoiled arrays in terms of populations of discrete nucleosomal states without considering the mechanics, structure, disorder and dynamics of the arrays.

**Figure 1.**
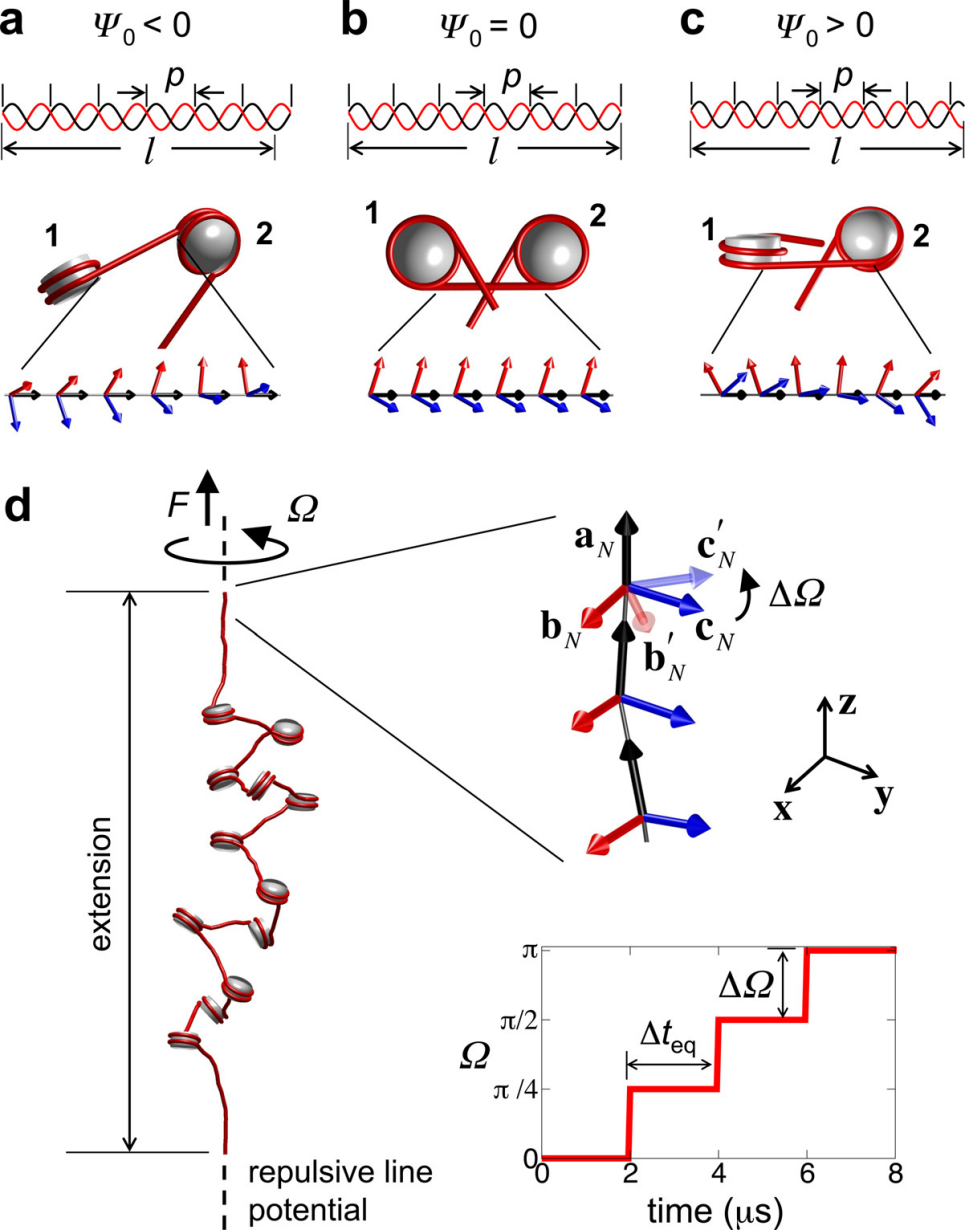
Rotational phase angle of nucleosomes and simulation setup. (**a**-**c**) Length and helical pitch of linker DNAs determines nucleosome phasing, and affects nucleosome topology: (**b**) *l* < *n*_b_*p* (where *n*_b_ is an integer, equal to 6 in this study) leads to counter-clockwise rotation of downstream nucleosome 2 with respect to upstream nucleosome 1 (*Ψ*_0_ < 0) leading to more open linker DNAs; (b) *l* = *n*_b_*p* leads to in-phase nucleosomes (*Ψ*_0_ = 0); and (c) *l* > *n*_b_*p* leads to clockwise rotation of 2 with respect to 1 (*Ψ*_0_ > 0) leading to more crossed linker DNAs. (**d**) Simulation setup for studying the torsional response of nucleosome arrays. External twisting Ω is applied by rotating the last linker DNA frame by angle ΔΩ in a step-wise manner every Δ*t*_eq_ while being subjected to a stretching force *F*. The repulsive line potential is drawn as dotted line and the DNAs and histone core are shown in red and gray, respectively.

Here, we provide the first detailed picture of the structure and dynamics of supercoiled nucleosome arrays through Brownian dynamics (BD) simulations of a coarse-grained (CG) model of nucleosome arrays that we have developed and validated against multiple types of experiments (26–28). The model captures the essential physics of chromatin folding, including the bending and twisting mechanics of linker DNAs, the excluded volume and DNA entry/exit geometry of nucleosomes, and the electrostatics of nucleosomes and linker DNAs. The BD simulations account for thermal fluctuations and viscous drag from solvent while computing the dynamics of each array component in the model. This approach allows us to obtain both the Boltzmann-distributed ensemble of array conformations at varying degrees of supercoiling and the real-time dynamics of DNA twist propagation along the arrays. To demonstrate how the intrinsic topology of arrays dictates their torsional behavior, we examine the effects of the phase angle *Ψ*_0_ defined as the *intrinsic* rotational angle between adjacent nucleosomes when the intervening linker DNAs are completely relaxed (undeformed) (29,30). As shown in Figure 1a–c, *Ψ*_0_ is determined by the ratio of the length *l* and average helical pitch *p* of the linker DNA, and it directly affects the nucleosomal entry/exit configuration of the linker DNAs, which in turn modulates the internal writhe of each nucleosome and the global structure of the arrays (31,32).

## MATERIALS AND METHODS

We investigate the torsional behavior of linker-histone deficient 12-nucleosome arrays with fixed *l* ≈ 62 bp linker DNAs flanked by two 135 bp-long linker DNAs, simulated at 10 mM monovalent salt and 310 K temperature (Figure 1d). This model system allows us to relate our computed extension-rotation curves to those obtained from single-molecule twisting experiments (21,22) on *in vitro* reconstituted, defined nucleosome arrays with similar linker lengths and studied under similar salt conditions. Furthermore, it is well known that under such conditions the arrays exhibit a beads-on-a-string conformation (33), which allows us to capture the fundamental mechanics of nucleosome arrays in response to torsional stresses without interference from nucleosome stacking and linker histone binding. However, it should be noted that the chromatin *in vivo* may not necessarily exhibit such an unfolded conformation and is likely to be present in more compact conformations, such as the 10-nm stacked-nucleosome fiber or the zigzag or solenoid 30-nm fiber (34).

The arrays are described using our CG model (26) in which each nucleosome is treated as a charged rigid cylinder and the linker DNAs are treated as charged bead-chains, each bead representing a 3 nm-long segment of DNA with *p* ≈ 10.4 bp. The linker DNA bead-chains are connected to the nucleosome cylinders at sites consistent with the entry/exit points of DNA in the nucleosome crystal structure. The linker DNA conformation, and the position and orientation of the nucleosomes, are described in terms of position vectors **r** and orthonormal unit vector frames {**a**, **b**, **c**} assigned to each linker DNA bead and nucleosome attachment site, where **a**, **b** and **c** represent the tangent, binormal and normal vectors, respectively (28). The total energy of the array includes contributions from: stretching, bending and twisting of the linker DNAs; electrostatic and excluded volume interactions of the nucleosome and linker DNA beads and constraints imposed on the end linker DNAs as described below.

To twist the array, the position and frame of the first linker DNA bead is held fixed and the frame of the last linker DNA bead is rotated step-wise in ΔΩ ≡ ± 45° increments every 2 μs while being pulled with a force of 0.34 pN in the *z*-direction, similar to experiments (21,22). Using this procedure, up to 12 and 10 turns of positive and negative supercoiling are introduced into the arrays, respectively. To prevent supercoiling of the end linker DNAs, they are subjected to a harmonic potential that suppresses their deviation away from the *z*-axis. We also introduce a repulsive line potential to prevent the array from crossing over the ends to conserve the array's topology during simulations. To investigate the effect of nucleosome phasing on array supercoiling, we generate arrays with different *Ψ*_0_ in the range −400° to 200°. This is achieved by altering the equilibrium twist angle between the vector frames of adjacent linker DNA beads, which is equivalent to varying the pitch *p* of the linker DNA, keeping their lengths *l* fixed (Figure 1a–c). The dynamics of the arrays are obtained using BD simulations. All results presented here represent ensemble averages over 50 independent twisting simulations at each *Ψ*_0_ value.

The CG model, BD simulations and twisting protocol are described in more detail in the Supplementary Text, Supplementary Figures S1 and S2 and Supplementary Table S1.

## RESULTS

### Nucleosome array conformations before twisting

**Figure 2.**
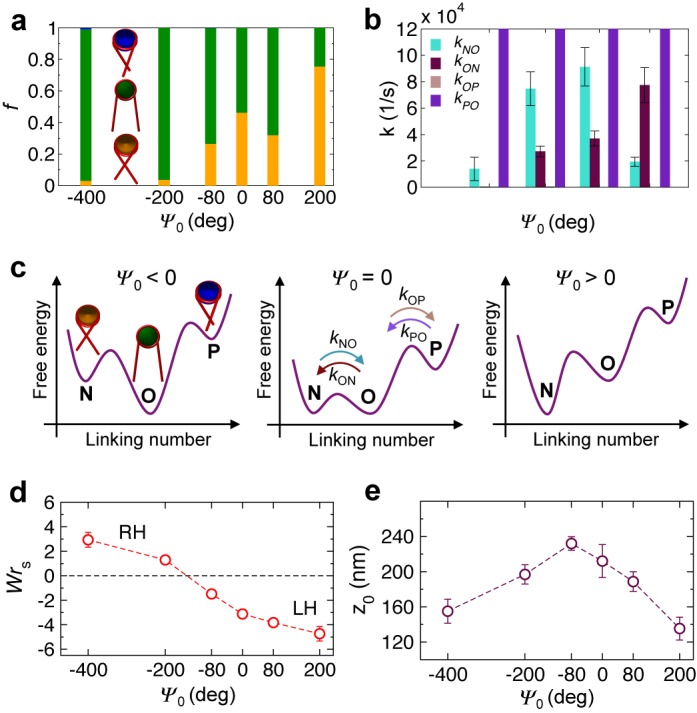
*Ψ*_0_-dependent array conformations before twisting. (**a**) Proportions of negative (orange), open (green) and positive (blue) nucleosome states and (**b**) rate constants of transitions across the three states as a function of *Ψ*_0_. For all *Ψ*_0_, *k*_PO_ are significantly larger than the axis limit and *k*_OP_ are essentially zero within SD. (**c**) Proposed energy landscape showing the free energies of the three nucleosome states and the barriers in between for three different regimes of *Ψ*_0_ as a function of nucleosomal linking number. (**d**) Array writhe *Wr*_S_ (RH and LH refer to right- and left-handed arrays) and (**e**) extension *z*_0_ as a function of *Ψ*_0_. Error bars in all plots indicate SD.

Before applying twist, we obtain the 0.34 pN tension-equilibrated conformations of nucleosome arrays as a function of the phase angle *Ψ*_0_. We first determine, using the procedure described in Supplementary Text and Supplementary Figure S1c, the fractions *f*_O_, *f*_N_ and *f*_P_ of nucleosomes in the array with open, negatively- and positively-crossed linker DNAs, respectively (Figure 2a). We refer to these states as *open*, *negative* and *positive*. The arrays are composed of mostly negative and open nucleosomes for all *Ψ*_0_, but their relative population varies strongly with *Ψ*_0_. When *Ψ*_0_ = 0, the nucleosomes are distributed almost evenly between open and negative states, consistent with recent FRET measurements (35). The nucleosomal DNA entry/exit angle of 120°, fixed in our model according to the nucleosome crystal structure, along with electrostatic repulsion between the entering and exiting linker DNAs likely promotes such a distribution. When *Ψ*_0_ < 0, the open state becomes more prevalent. In this case, the DNA pitch increases as the DNA is undertwisted. Consequently, the ‘downstream’ nucleosomes are oriented anticlockwise relative to the ones ‘upstream’, causing the two linkers DNAs to diverge (Figure 1a). When *Ψ*_0_ > 0, the negatively-crossed state becomes dominant. Here, the DNA pitch decreases as the DNA is overtwisted, leading to clockwise rotation of the downstream nucleosome, and subsequent convergence of the linker DNAs (Figure 1c).

To examine the rates of nucleosome transitions across the three states, we compute the first-order rate constant *k*_XY_ for each of the four transitions as the inverse of the average time a nucleosome resides in state X before transitioning to state Y (Figure 2b). We find that *k*_ON_ and *k*_NO_ are overall large and roughly comparable to each other at moderate values of *Ψ*_0_ (−80° and 80°), while they become small and differ significantly from each other at extreme values of *Ψ*_0_ (especially at −400°). In contrast, the other two rate constants remain extremely small (*k*_OP_) or extremely large (*k*_PO_) across all *Ψ*_0_. Thus, moderate phasing of nucleosomes seems to impose a weak ‘twisting constraint’ on the nucleosomes, allowing them to undergo frequent conformational fluctuations between the open and negative states, leading to irregular arrangement of nucleosomes in the arrays (Supplementary Figure S4). On the other hand, extreme phasing imposes a strong twisting constraint on the nucleosomes, causing their conformations to be strongly correlated with each other, leading to their more regular, helical arrangement in the arrays (Supplementary Figure S4).

The nucleosomal state populations and transition rates inform us on the relative free energies of the states and the energy barriers between them, respectively (Figure 2c). At moderate *Ψ*_0_, the open and negative states are almost equally distributed, suggesting similar free energies of the two states. Frequent transitions between the two states further suggest a relatively small energy barrier between them. However, for extremely negative (positive) *Ψ*_0_, the open (negative) state becomes more prevalent, suggesting a lowering (raising) of the free energy of the open state relative to that of the negative state. Moreover, infrequent transitions between the two states at these extreme *Ψ*_0_ suggests the appearance of larger energy barriers between the two states, giving rise to the strong twisting constraint discussed above. The small proportion of positive nucleosomes, even at the extremely negative *Ψ*_0_, implies that the positive state has much higher free energy than the open and negative states.

To characterize the topology of the arrays, we compute their writhe. Traditionally, DNA writhe *Wr* characterizes the number of times the DNA axis crosses itself averaged over all 2D projections. When applied to arrays, *Wr* would include contributions from wrapping of DNA in nucleosomes and from global coiling of arrays as determined by the path of the nucleosomes. Since our focus is on the latter, we calculate the writhe of the closed path generated by connecting the centers of nucleosomes with straight lines and connecting the array ends by a suitable curve (16), as described in Supplementary Text and Supplementary Figure S3. Such a writhe of the array ‘skeleton’, denoted by *Wr*_S_, allows us to characterize the handedness of the arrays, with *Wr*_S_ < 0 and *Wr*_S_ > 0 representing left- and right-handed coiling of the arrays, respectively. The computed *Wr*_S_ (Figure 2d) along with visualization of the arrays (Supplementary Figure S4) indicates that the arrays form right-handed zigzags at very negative *Ψ*_0_. As *Ψ*_0_ increases, the arrays become more uncoiled, as noted by the decrease in *Wr*_S_, until at *Ψ*_0_ ≈ −140°, the arrays are largely uncoiled. Further increase in *Ψ*_0_ leads to the formation of left-handed solenoids. An explanation for the appearance of left- and right-handed structures depending on the sign of *Ψ*_0_ is provided in Supplementary Figure S5.

Lastly, we obtain the *Ψ*_0_-dependence of the extension *z*_0_ of the arrays (Figure 2e), where *z*_0_ is the projected end-to-end distance of the array along *z*-axis. We find that *z*_0_ exhibits a bell-shaped curve with the maximum occurring at *Ψ*_0_ ≈ −80°. The short *z*_0_ at extreme *Ψ*_0_, both negative and positive, evidently occurs due to the strong coiling of the arrays, as indicated by large *Wr*_S_ (Figure 2d). Moderate *Ψ*_0_ lead to lesser coiling of the arrays, leading to larger *z*_0_. Here, the nucleosomes can undergo rapid transition between open and negative states (Figure 2c), leading to an irregular structure of the arrays with a low level of supercoiling that can be stretched easily with an external force. Interestingly, the least coiled state of the arrays occurs at negative values of *Ψ*_0_, close to the value at which the arrays have the largest extension.

### Extension-rotation behavior

**Figure 3.**
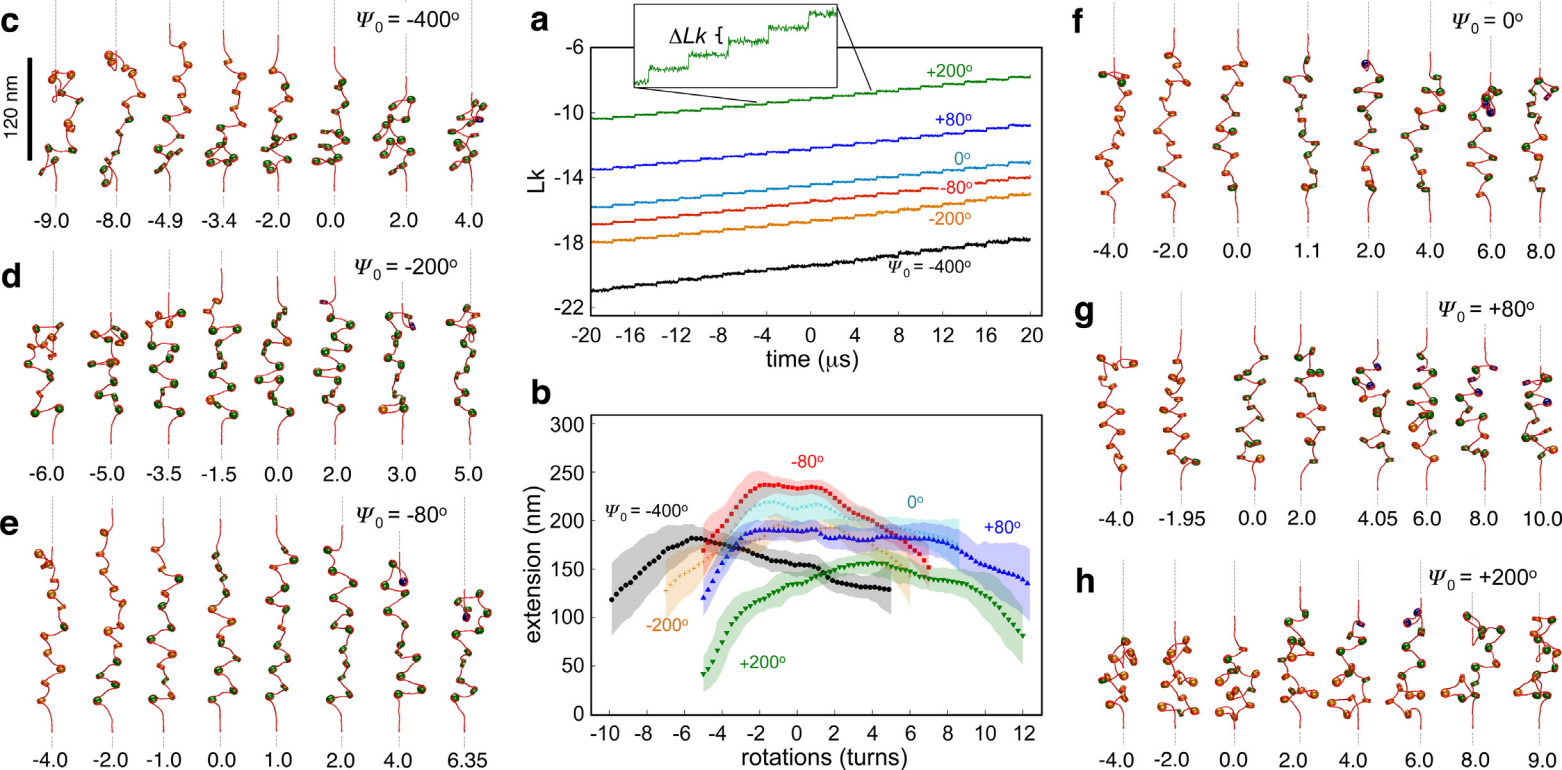
*Ψ*_0_-dependent torsional response of nucleosome arrays. (**a**) Time evolution of the linking number and (**b**) extension-rotation curves of arrays with different *Ψ*_0_. Shaded regions represent standard deviations. (**c**–**h**) Representative conformations of arrays during twisting for different *Ψ*_0_ values. Numbers at the bottom indicate the number of imposed turns *n*. *SI movies* provide a higher time resolution of these conformational changes.

We next twist the arrays in the positive and negative directions, starting from the untwisted arrays equilibrated under the stretching force. The amount of twist introduced into the arrays is quantified in terms of the number of turns *n* by which the free end of the array is rotated. To verify that no twist is being lost through the ends, we measure the linking number *Lk* of the arrays as a function of time. *Lk* is computed as the sum of twist *Tw* and writhe *Wr*, as detailed in the Supplementary Text. Figure 3a displays the *Lk* profiles for all tested values of *Ψ*_0_. In general, *Lk* shows a step-wise increase after each end rotation, and the step size measures the linking number difference Δ*Lk* given by the sum of the changes in the writhe and twist of the array Δ*Lk =* Δ*Wr +* Δ*Tw.* For all *Ψ*_0_, the measured Δ*Lk* fall in the range 0.1–0.13, which agrees well with the amount of twist ΔΩ/2π = 0.125 introduced into the arrays through each end-rotation step. These results confirm that the topology of the arrays is conserved during twisting. The negative values of *Lk* result from the ∼1.65 left-handed superhelical turns of DNA wrapped in nucleosomes. The separation between *Lk* profiles for different *Ψ*_0_ points to its effects on the array topology, as described earlier. Supplementary Table S2 lists the measured *Lk* for all *Ψ*_0_ and its partitioning into *Tw* and *Wr* at *n* = 0.

The torsional response of the arrays is characterized in terms of changes in their extension with respect to imposed rotations *n* (Figure 3b). Though the extension-rotation curves for all *Ψ*_0_ exhibit a bell-shaped profile with a single peak, similar to those measured experimentally (21,22), they differ appreciably from each other in symmetry and peak location. Specifically, as *Ψ*_0_ becomes increasingly negative, the extension peak shifts toward negative *n* whereas it shifts toward positive *n* as *Ψ*_0_ becomes increasingly positive. For instance, at *Ψ*_0_ = −400° and 200°, the curves show sharp peaks at *n* ≈ −6 and 4 turns, respectively. Strikingly, for moderate values of *Ψ*_0_ (−80°, 0° and 80°) the peak broadens, and an intermediate regime opens up, where extension remains almost constant. In fact, for *Ψ*_0_ = 80°, this plateau extends beyond 10 turns and is shifted toward positive rotations.

**Figure 4.**
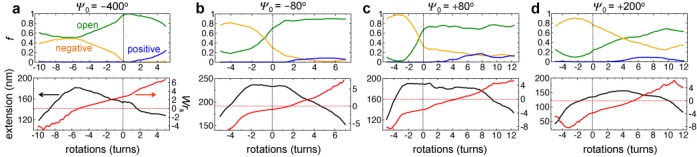
Local and global conformational changes in arrays under external twisting. (Top panel) Dynamic proportions of negative (orange), open (green) and positive (blue) nucleosomal states as a function of imposed rotations *n* for four different *Ψ*_0_ values. (Bottom panel) Corresponding plots of the extension (black) and *Wr*_S_ (red) of arrays.

To uncover the underlying mechanism behind this unique *Ψ*_0_-dependent extension-rotation behavior, we trace the proportions *f*_O_, *f*_N_ and *f*_P_ of nucleosomes in the open, negative and positive states, and also compute the writhe *Wr*_S_ of the arrays as a function of *n* (Figure 4), which quantify respective changes in the nucleosomal and global writhe of the arrays. One expects negative twisting (*n* < 0) to favor nucleosomal states and array conformations that possess the most negative writhe, i.e., negative state and left-handed arrays, while positive twisting (*n* > 0) is expected to favor states and conformations with the most positive or least negative writhe, i.e., positive state and right-handed arrays (Supplementary Figure S6).

We begin by explaining the negative shift in the extension-rotation curve for *Ψ*_0_ = −400° arrays (Figure 4a). These arrays exhibit small extension, right-handed zigzag conformation and mostly open nucleosomes *before* twisting (Figure 2). Upon *positive* twisting, the arrays undergo further positive coiling, as noted by the increase in *Wr*_S_, leading to the observed decrease in array extension. The existence of a large energy barrier between the positive and open states (Figure 2c) prevents nucleosomes from flipping into positive states, as noted by the minimal increase in *f*_P_, even for large *n*. Upon *negative* twisting, the nucleosomes undergo transitions from the open to the negative state, which are separated by moderate energy barrier, and the arrays uncoil, as noted by the decrease in *Wr*_S_, leading to the observed increase in array extension upon negative twisting. By *n* ≈ −6 turns, the arrays are largely uncoiled, as noted by *Wr*_S_ ≈ 0, and the array extension is at its greatest. Additional negative twisting beyond this point destabilizes the arrays, triggers their negative coiling, as noted by *Wr*_S_ becoming increasingly negative, which leads to the observed decrease in array extension.

The positive shift in *Ψ*_0_ = 200° arrays can also be understood using similar arguments (Figure 4d). Here, the *untwisted* arrays exhibit left-handed solenoidal conformations and comprise of mostly negative nucleosomes (Figure 2). *Positive* twisting causes nucleosomes to transition from negative to open states, which are separated by a moderate energy barrier, and to uncoiling of the arrays, resulting in an increase in their extension. By *n* ≈ −4 turns, the arrays are completely uncoiled and they exhibit the largest extension. Further positive twisting now leads to right-handed coiling, causing the extension to decrease beyond this point. In contrast, *negative* twisting leads to further negative coiling of the already negatively coiled arrays, leading to the observed decrease in array extension. Note that the nucleosomes cannot undergo any more transitions as most of them are already in the negative state. Thus, the location of the extension-rotation peaks for extreme values of *Ψ*_0_, i.e. toward negative or positive *n*, is greatly affected by the handedness of the arrays’ initial conformation.

To explain the existence of the broad plateau in the extension-rotation curves of arrays with *Ψ*_0_ = −80°, 0° and −80°, we recall that the *untwisted* arrays at these *Ψ*_0_ exhibit a mixture of open and negative states separated by a small energy barrier. Consequently, the arrays can rapidly relax their torsional stress through nucleosomal transitions between the open and negative states without the need for global coiling of the arrays, leading to the extension plateau during negative and positive twisting. Only after saturation of the negative or open states, depending on the twisting direction, do the arrays begin to supercoil more strongly, leading to the observed decrease in extension beyond the plateau region. Both these effects can be observed in Figure 4b and c, i.e. the slow variation in writhe *Wr*_S_ and fast variation in state populations *f*_N_ and *f*_O_ within the plateau and the faster variation in *Wr*_S_ and saturation of *f*_N_ and *f*_O_ outside the plateau.

We also note that, in spite of the same magnitude of *Ψ*_0_, the plateau for *Ψ*_0_ = 80° arrays is much broader than that for *Ψ*_0_ = −80° arrays, especially on the *n* > 0 side. This can be understood by noting that the former arrays exhibit left-handed zigzag conformations (*Wr*_S_ ≈ −3) before twisting. These arrays then require extensive positive twisting (*n* ≈ 7) to trigger a substantial decrease in their extension, as the twisting has to not only convert all negative nucleosomes into open nucleosomes but also completely unwind the left-handed conformation of the arrays, before the arrays start to positively coil. Moreover, these arrays seem to show a greater propensity to form positive nucleosomes, which further delays positive coiling of the arrays. The latter arrays, on the other hand, are already fairly uncoiled (*Wr*_S_ ≈ −1) before twisting, and hence begin to supercoil immediately following the saturation of open nucleosome states at *n* ≈ 2.

### Propagation of DNA twist and torsional resilience

To investigate how torsional stress (DNA twist) propagates along the array during twisting, we compute the autocorrelation *C*(*t*,*i*) = 〈**c***_i_*(0)·**c***_i_*(*t*)》 between the normal vector **c***_i_* of each linker DNA segment (bead) and nucleosomal attachment site *i before* and at time *t after* application of twist, where the angular brackets represent ensemble average over multiple simulation runs at each *Ψ*_0_. *C*(*t*,*i*) allows us to detect in real time the passage of DNA twist along the array, as *C*(*t*,*i*) ≈ 1 indicates DNA segments that are yet to undergo twisting, while *C*(*t*,*i*) < 1 indicates segments that have undergone twisting.

**Figure 5.**
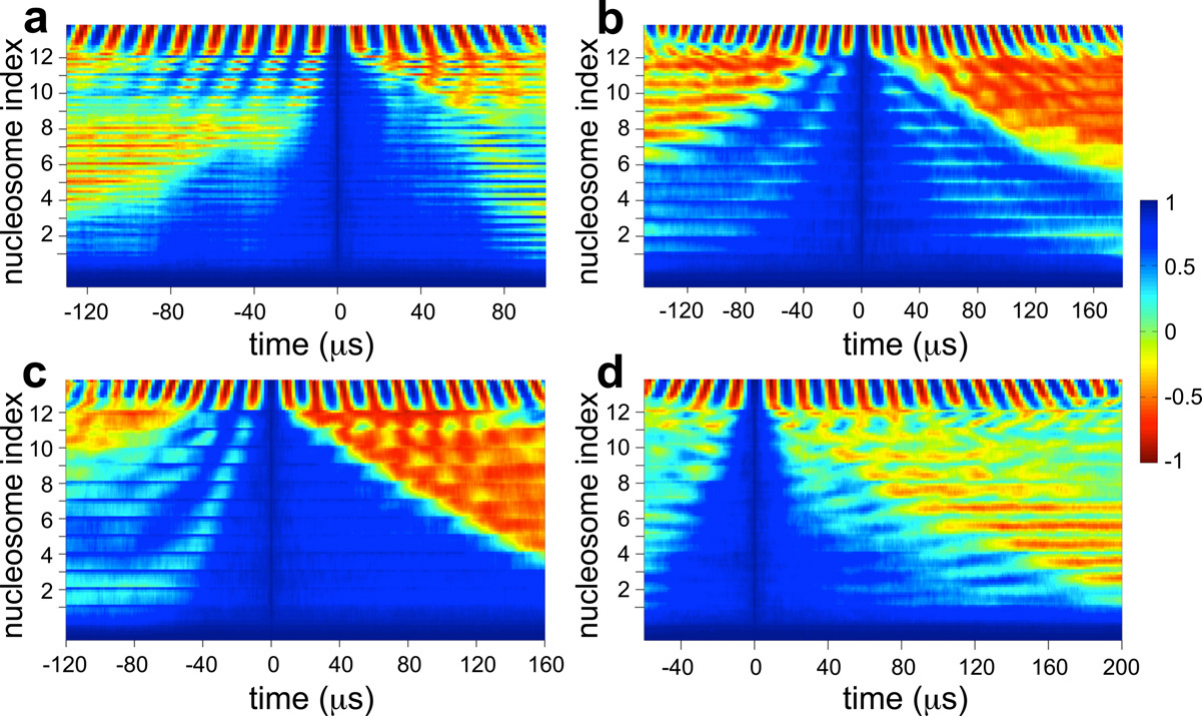
DNA twist propagation along arrays. *C*(*t,i*) landscape of arrays with (**a**) *Ψ*_0_ = −400°, (**b**) −80°, (**c**) 80° and (**d**) 200°. The location of each nucleosome in the array is marked on the *y*-axis; the end linker DNA being twisted is attached to the last nucleosome 12, and *t* = 0 corresponds to arrays at the onset of twisting. Sixteen microsecond represents 1 rotation.

Figure 5 displays the *C*(*t*,*i*) landscape for arrays with different *Ψ*_0_. The last few DNA segments in all arrays exhibit oscillations in *C*(*t*,*i*) between values of 1 and −1, indicative of the continuous twisting of the terminal linker DNA. The DNA twist ‘front’ is observed as the boundary of the blue region encompassing the *t* = 0 line. The left- and right-hand boundaries represent front propagation during negative and positive twisting, respectively, and their slopes represent speeds of twist propagation. We observe a dramatic effect of *Ψ*_0_ on twist propagation. Extreme values of *Ψ*_0_ lead to fast but abrupt propagation of DNA twist, which occurs in sharp bursts interspersed with long pauses (Figure 5a and d). The fast bursts arise from strongly correlated flipping of nucleosomes, allowing quick propagation of DNA twist from one linker DNA to the next, while the long pauses occur due to global coiling of arrays when nucleosomes are unable to flip due to steric constraints. Also, the speed of twist propagation differs for positive versus negative twisting, with higher speeds occurring during positive twisting for *Ψ*_0_ = −400°, and *vice versa* for *Ψ*_0_ = 200°. Moderate *Ψ*_0_, on the other hand, lead to slow but uniform twist propagation as well as similar speeds of propagation during positive and negative twisting (Figure 5b and c). The slower speeds likely occur due to transitions between open and negative nucleosomes. We also note a step-like propagation of twist across linker DNAs, indicating the existence of energy barriers during flipping of nucleosomes. Interestingly, the speed of twist propagation of ∼10 Mbp/s, even for the slowest scenario (*Ψ*_0_ = −80°), is quite rapid.

**Figure 6.**
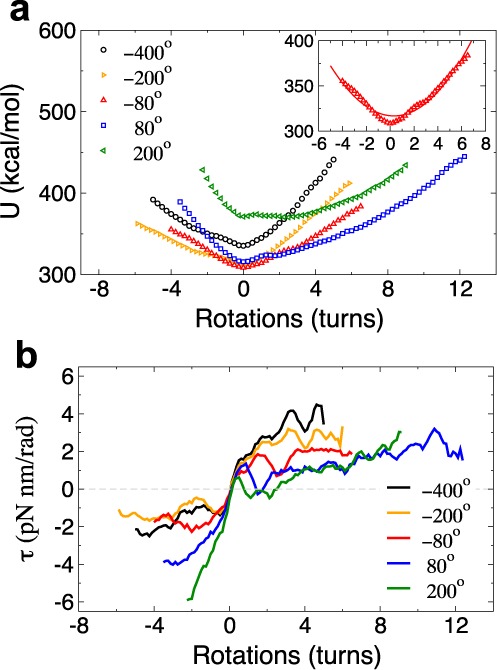
Torsional resilience of nucleosome arrays. (**a**) Total energy and (**b**) torque profiles of arrays with different *Ψ*_0_ as a function of the number of rotations. The insets show the energies for *Ψ*_0_ = 80° fitted to a harmonic function.

To obtain the torsional rigidity *C*_array_ of the arrays, we examine their total energy as a function of imposed rotations (Figure 6a). The energy profiles exhibit a minimum located at *n* ≈ 0 regardless of *Ψ*_0_. Fitting this energy profile to a harmonic function yields *C*_array_ = 0.3 kcal/mol nm for arrays with *Ψ*_0_ = −80° (Figure 6a; Supplementary Text). We also estimate the torque experienced by the arrays as a function of imposed rotations (see Supplementary Text) and find that it remains <5 pN nm/rad for a broad range of rotations (Figure 6b), i.e. slightly smaller than the torque exerted by the RNA polymerase enzyme (36) and much smaller than that required to denature DNA (17). The small torsional rigidity of the arrays as compared to that of naked DNA (*C*_DNA_ = 57 kcal/mol nm (14)) and the small torques experienced by the arrays confirm the high torsional resilience of chromatin observed in experiments (21,22). We note that *C*_array_ is largely independent of *Ψ*_0_, but is likely affected by other properties like the length of the linker DNAs and their entry/exit angle at nucleosomes. Another important parameter is the number of nucleosomes in the array, as one expects longer arrays with more combinations of nucleosome states to accommodate larger amount of DNA twist.

## DISCUSSION

We have elucidated the torsional behavior of nucleosome arrays using an approach that not only captures the macroscopic response of the arrays but also the microscopic structure and dynamics of the arrays during twisting. We uncover a striking dependence of the arrays’ torsional response on the intrinsic rotational phase angle *Ψ*_0_ between adjacent nucleosomes. Extreme values of *Ψ*_0_ lead to asymmetric extension-rotation curves with maxima shifted by negative or positive rotations depending on the sign of *Ψ*_0_ and to fast, irregular propagation of DNA twist. On the other hand, moderate *Ψ*_0_ values yield more symmetric extension-rotation curves with broad maxima and slow, uniform twist propagation. The torsional rigidity of arrays is largely unaffected by *Ψ*_0_ and is found to be significantly smaller than that of DNA. Analyses of array conformations reveal the underlying mechanism responsible for the observed torsional response and its strong *Ψ*_0_-dependence. Specifically, *Ψ*_0_ prescribes the energy landscape of the nucleosome's topological states, and changes in *Ψ*_0_ alter the population of nucleosomes in each state as well as the folded geometry and topology of the arrays. This affects the local and global modes of torsional stress relaxation available to the arrays during twisting, leading to the observed *Ψ*_0_-dependent torsional responses. These results have important implications.

First, they reconcile the discrepancy between single-molecule twisting measurements on nucleosome arrays by two different groups, where one group observed the extension-rotation peak shifting toward negative rotations (21) while the other noted the exact opposite (22). Judging from similar hysteretic behavior observed in both experiments, the discrepancy cannot be attributed to different definitions of positive and negative twisting. Based on our findings (Figure 3b), we propose that the arrays employed in the two sets of experiments likely exhibited different topologies, with those exhibiting negative shift possessing mostly open nucleosomes and *Ψ*_0_ < 0, while those exhibiting positive shift possessing predominantly negative nucleosomes and *Ψ*_0_ > 0. These topological differences likely arose during array reconstitution and manipulation, but could not have occurred due to differences in linker histone binding, which can alter the configurations of linker DNAs at nucleosomes (28), as these proteins were absent in both experiments.

Second, the sensitivity of extension-rotation curves to nucleosome phasing observed in this study suggests a plausible method for experimentally characterizing the phase angle *Ψ*_0_ of nucleosome arrays based on their torsional response. Since *Ψ*_0_ is a function of linker DNA length and DNA helical pitch, the quantitation of *Ψ*_0_ could lead to the estimation of one of these quantities if the other was already known. Such a torsion-based assay could also provide valuable information on the nucleosome entry/exit conformation of linker DNAs and differentiate between left- and right-handed coiling of nucleosome arrays, due to their dependence on *Ψ*_0_ (Figure 2c and d). Indeed, the internal structure of chromatin fibers, especially the linker DNA geometry at nucleosomes, remains far from fully resolved.

Third, our results can be used to predict the effects of fluctuations in the amount of DNA wrapped within nucleosomes. In particular, the crystal structure of the nucleosome core shows that it can accommodate ±1–2 bp changes in wound DNA (37), which is important for biological functions like nucleosome remodeling. Therefore, the accommodation of an additional 2 bp of DNA into nucleosomes, which is expected to shorten the linker DNA by the same amount, should lead to ∼72° anticlockwise rotation of the nucleosome (32), i.e. a phase angle change Δ*Ψ*_0_ ∼ −72°, assuming that the DNA entry/exit angle remains the same. Our simulations suggest that this phase angle shift should lead to a stabilization of the open state of the nucleosome (Figure 2a and c), reduction in the degree of left-handed supercoiling of the arrays (Figure 2d), and a slight negative shift in the extension-rotation curve (Figure 3b). Conversely, a release of 2 bp of DNA from nucleosomes should lead to stabilization of the negatively crossed nucleosomal state, increase in array supercoiling, and positive shift and broadening of the extension-rotation curve.

Fourth, the strong dependence of the global structure of nucleosome arrays and their torsional response on the entry/exit configuration of linker DNAs at nucleosomes (Figures 2d and 3b) suggests that chromatin organization can be easily modulated by processes that affect linker DNA conformation, either through changes in the phase angle *Ψ*_0_ or through changes in the amount of DNA wrapped in nucleosomes. The former could be modulated by nucleosome positioning (38), i.e. locations along genomic DNA at which nucleosomes form, and by changes in the helical pitch of DNA via salt conditions and protein binding. The latter could be modulated by histone modifications. For instance, the open linker DNA conformation is found in transcriptionally active chromatin and promoted by hyper-acetylated histones (39), while the crossed state is more stabilized by DNA methylation in inactive chromatin (40).

Although we have not directly examined the effects of changes in the linker DNA entry/exit angle (due to thermal fluctuations, external forces, or changes in salt conditions), it may be possible to predict these effects based on our results. Specifically, a decrease in the entry/exit angle, corresponding to enhanced wrapping of DNA, should lead to stabilization of the negative nucleosome states, an effect akin to *Ψ*_0_ becoming positive (Figure 2a and c). Our simulations suggest that *Ψ*_0_ > 0 should cause the arrays to become more negatively coiled (Figure 2d) and compact (Figure 2e), and lead to a positive shift in the extension-rotation curves (Figure 3b). If the total length of DNA is conserved, the enhanced wrapping of DNA will also lead to a reduction in the length of the linker DNAs, causing a decrease in *Ψ*_0_. Our simulations suggest that *Ψ*_0_ < 0 should cause the arrays to become less negatively supercoiled (Figure 2d) and extended (Figure 2e) and lead to a negative shift in the extension-rotation curves (Figure 3b). Thus, when the total length of DNA is fixed, the conformation and torsional response of the arrays to enhanced wrapping will depend on the relative magnitudes of these two opposing effects, though which effect becomes dominant is difficult to predict, and could form the basis for future studies.

Fifth, the twisting-induced changes in the structure of nucleosome arrays studied here could play important roles in chromatin assembly and gene regulation. For instance, torsionally stressed DNA might play an important role at early stages of chromatin organization in bringing relevant nucleosomes into a tentative trajectory, which are then stabilized by the linker histone and histone tails. Our results suggest that torsional stresses could lead to further opening or crossing of the linker DNAs (Figure 4) and to further extension or compaction of the chromatin fiber (Figure 3b), depending on twisting direction and phase angle *Ψ*_0_. Such local and global modulation of chromatin structure affects the accessibility of DNA for interacting with regulatory proteins like transcription factors. These roles might partially explain the negative correlation observed between linker DNA length and gene activity (32), as short linker DNAs develop higher twist densities compared to longer ones for equivalent amount of twisting.

Lastly, it was recently shown that torsional stresses generated at one genomic location could melt twist-sensitive DNA sequences much further upstream (3) and that accumulation of torsional stress, elevated through topoisomerase inhibition, could destabilize nucleosomes downstream (41). Our results in Figure 5 indicate that such propagation of DNA twist occurs at an extremely fast rate (*v* ≥ 10 Mbp/s), capable of transmitting signals along the DNA at much faster rates than achievable through diffusion of chemicals. As an example, consider a portion of chromatin fiber about 100 nm long that packages *l* ∼10 kbp of DNA. If a ‘chemical’ signal were to diffuse along the length of the DNA, it would take a time on the order of *l*^2^/3*D* ≈ 33 ms (42), where *D* ∼10^6^ bp^2^/s is the typical 1D diffusivity of a signaling protein along DNA (43). In contrast, a ‘mechanical’ signal, via twist propagation, would take orders of magnitude less time *l*/*v* ∼100 μs to travel the same length of DNA. Thus, twist propagation could be an efficient route for gene regulation, and future studies should reveal the universality of such a regulatory strategy.

## SUPPLEMENTARY DATA

Supplementary Data are available at NAR Online.

SUPPLEMENTARY DATA
