# Supplementary material for: Torsional behavior of chromatin is modulated by rotational phasing of nucleosomes
Source: Nucleic Acids Res. 2014 Aug 6;42(15):9691–9. doi: 10.1093/nar/gku694 (PMC4150795; doi:10.1093/nar/gku694)
Supplement: SUPPLEMENTARY DATA [file supp_42_15_9691__index.html]

Torsional behavior of chromatin is modulated by rotational phasing of nucleosomes — Torsional behavior of chromatin is modulated by rotational phasing of nucleosomes — SUPPLEMENTARY DATA 

# Torsional behavior of chromatin is modulated by rotational phasing of nucleosomes

## SUPPLEMENTARY DATA

**Files in this Data Supplement:**

- SUPPLEMENTARY DATA
- SUPPLEMENTARY DATA
- SUPPLEMENTARY DATA
- SUPPLEMENTARY DATA
- SUPPLEMENTARY DATA
- SUPPLEMENTARY DATA
- SUPPLEMENTARY DATA
- SUPPLEMENTARY DATA
