# Supplementary material for: Torsional behavior of chromatin is modulated by rotational phasing of nucleosomes
Source: Nucleic Acids Res. 2014 Aug 6;42(15):9691–9. doi: 10.1093/nar/gku694 (PMC4150795; doi:10.1093/nar/gku694)
Supplement: SUPPLEMENTARY DATA [file supp_gku694_nar-01019-m-2014-File008.pdf]

Supplementary Information

Gi-Moon Nam and Gaurav Arya

Department of NanoEngineering, University of California at San Diego,
9500 Gilman Drive, La Jolla, CA 92093-0448

1 Coarse-grained (CG) model of nucleosome arrays

In our CG model, each nucleosome, inclusive of the histone core and the ∼1.65-turn wrapped DNA, is constructed from $n_c = 26$ charged beads, each of size $\sigma_c = 2.0$ nm (Fig. S1a). The beads are arranged in three stacks, where the top and bottom stacks each contain 9 beads evenly placed along a circumference of radius $r_o = 4.30$ nm and the middle stack contains 8 beads evenly placed along a circumference of radius $r_i = 2.64$ nm. The relative positions of these beads are held fixed to treat the entire nucleosome as one rigid body. Each linker DNA is modeled as a discretized wormlike chain composed of six charged beads to mimic the 62-bp linkers of chicken erythrocyte chromatin, where each bead represents a $l_0 = 3$ nm-long strand of relaxed B-DNA. Each nucleosome is attached to two linker DNAs, termed "entry" and "exit" linkers, at sites consistent with the 1KX5 crystal structure of the nucleosome [1] subtending an entry/exit angle of $\theta_0 = 120°$ and separated by a distance of $2w_0 = 3.6$ nm along the nucleosome axis (Fig. S1a and b). We do not explicitly model histone tails, as we examine the arrays under low salt conditions, similar to twisting experiments [2, 3]. Under these conditions, the arrays exhibit unfolded conformations and histone tail interactions become relatively unimportant [4].

The model geometry is shown in Fig. S1b. The positions of linker DNA beads and nucleosome centers are represented by vectors $\mathbf{r}_i$ ($i = 1, ..., N$), where N is the total number of linker DNA beads and nucleosomes in the array. The positions of linker DNA entry and exit sites at nucleosomes i are described by vectors $\mathbf{r}_i^-$ and $\mathbf{r}_i^+$, respectively. The orientation of the nucleosomes and the linker DNA beads are described by the orthonormal unit vectors frame $\{\mathbf{a}_i, \mathbf{b}_i, \mathbf{c}_i\}$, where $\mathbf{a}_i$ is the tangent vector, and $\mathbf{b}_i$ and $\mathbf{c}_i$ are the binormal and normal vectors, respectively [5]. When index i represents a nucleosome, the vectors $\mathbf{a}_i$ and $\mathbf{b}_i$ lie in the plane of the nucleosome, where $\mathbf{a}_i$ points along the tangential direction of the wrapped DNA at the nucleosomal exit site, $\mathbf{b}_i$ points towards the nucleosome center from the exit site, and $\mathbf{c}_i = \mathbf{a}_i \times \mathbf{b}_i$. The entry/exit site positions $\mathbf{r}_i^-$ and $\mathbf{r}_i^+$ can be obtained in terms of $\mathbf{r}_i$, $\mathbf{a}_i$, $\mathbf{b}_i$, $\mathbf{c}_i$, w_0, and θ_0 from geometry. The orientation of linker DNA exiting nucleosome i is described by the vector frame $\{\mathbf{a}_i^{\rm DNA}, \mathbf{b}_i^{\rm DNA}, \mathbf{c}_i^{\rm DNA}\}$, where $\mathbf{a}_i^{\rm DNA}$ points along the vector connecting the nucleosomal exit site to the first bead of the exiting linker DNA, $\mathbf{b}_i^{\rm DNA}$ is the binormal vector, and $\mathbf{c}_i^{\rm DNA} = \mathbf{a}_i^{\rm DNA} \times \mathbf{b}_i^{\rm DNA}$. We define two additional vector frames $\{\mathbf{a}_i^-, \mathbf{b}_i^-, \mathbf{c}_i^-\}$ and $\{\mathbf{a}_i^+, \mathbf{b}_i^+, \mathbf{c}_i^+\}$ to describe the orientation of the wrapped DNA at the entry and exit sites of nucleosome i, respectively. The vector $\mathbf{a}_i^-$ points along the tangential direction of the wrapped DNA at the nucleosomal entry site and is a geometrical function of $\mathbf{a}_i$, $\mathbf{b}_i$, and θ_0, while $\mathbf{b}_i^-$ points towards the nucleosome center from the entry site, and $\mathbf{c}_i^- = \mathbf{c}_i$. Since $\{\mathbf{a}_i, \mathbf{b}_i, \mathbf{c}_i\}$ are defined according to the nucleosome exit site, the frame $\{\mathbf{a}_i^+, \mathbf{b}_i^+, \mathbf{c}_i^+\} = \{\mathbf{a}_i, \mathbf{b}_i, \mathbf{c}_i\}$. When i represents a linker DNA bead, $\mathbf{a}_i$ defines the unit vector connecting linker bead i to either the next linker DNA bead $i + 1$ or the entry site on nucleosome $i + 1$, $\mathbf{b}_i$ represents the binormal vector, and $\mathbf{c}_i = \mathbf{a}_i \times \mathbf{b}_i$.

The bending and twisting of the linker DNAs is determined in terms of Euler angles $\{\alpha_i, \beta_i, \gamma_i\}$ describing the relative orientations of adjacent coordinate frames. Specifically, when both i and $i + 1$ represent

linker DNA beads, then $\{\alpha_i, \beta_i, \gamma_i\}$ describes the transformation $\{\mathbf{a}_i, \mathbf{b}_i, \mathbf{c}_i\} \to \{\mathbf{a}_{i+1}, \mathbf{b}_{i+1}, \mathbf{c}_{i+1}\}$. When i represents a linker DNA bead and $i+1$ represents a nucleosome, then $\{\alpha_i, \beta_i, \gamma_i\}$ describes the transformation $\{\mathbf{a}_i, \mathbf{b}_i, \mathbf{c}_i\} \to \{\mathbf{a}^-_{i+1}, \mathbf{b}^-_{i+1}, \mathbf{c}^-_{i+1}\}$. When i represents a nucleosome and $i+1$ represents a linker DNA, then $\{\alpha_i, \beta_i, \gamma_i\}$ describes the transformation $\{\mathbf{a}^{\mathrm{DNA}}_i, \mathbf{b}^{\mathrm{DNA}}_i, \mathbf{c}^{\mathrm{DNA}}_i\} \to \{\mathbf{a}_{i+1}, \mathbf{b}_{i+1}, \mathbf{c}_{i+1}\}$. We introduce an additional set of Euler angles $\{\alpha^+_i, \beta^+_i, \gamma^+_i\}$ to describe the transformation $\{\mathbf{a}^+_i, \mathbf{b}^+_i, \mathbf{c}^+_i\} \to \{\mathbf{a}^{\mathrm{DNA}}_i, \mathbf{b}^{\mathrm{DNA}}_i, \mathbf{c}^{\mathrm{DNA}}_i\}$. Readers are referred to the Supplementary Material of Ref. [5] for a more extensive description of orientation vector frames and Euler angles.

The total potential energy U of a nucleosome array is given by terms describing the stretching, bending, and twisting of the linker DNAs and the excluded volume and electrostatic interactions of the linker DNA and nucleosome beads. The array potential energy also contains contributions from confining and repulsive line potentials during twisting whose basis and implementation is described further below.

The stretching energy U_{str} of linker DNAs is given by

$$U_{\mathrm{str}} = \frac{A}{2} \sum_{i=1}^{N-1} (l_i - l_0)^2, \tag{S1}$$

where A is the stretching modulus, l_i is the distance between two adjacent linker DNA beads or that between nucleosome entry/exit sites and the connected linker DNA beads, and l_0 is the corresponding equilibrium distance for unstretched DNA.

The linker DNA bending energy U_{bend} is given by

$$U_{\mathrm{bend}} = \frac{B}{2} \left[\sum_{i=1}^{N-2} (\beta_i)^2 + \sum_{i=1}^{N_{\mathrm{c}}} (\beta^+_i)^2 \right], \tag{S2}$$

where B is the bending modulus, β_i is a Euler angle describing the angle between three consecutive linker DNA beads, and N_{c} is the number of nucleosomes in the array.

The linker DNA twisting energy U_{tw} is given by

$$U_{\mathrm{tw}} = \frac{C}{2l_0} \sum_{i=1}^{N-2} (\alpha_i + \gamma_i - \phi_0)^2, \tag{S3}$$

where C is the torsional modulus of DNA, $\alpha_i + \gamma_i$ is the twist angle between adjacent DNA frames, and ϕ_0 is the intrinsic (equilibrium) twist angle between the frames for relaxed DNA. For $\phi_0 = 0$, the twisting energy is minimized when $\alpha_i + \gamma_i = 0$ for all linker DNA frames. This places adjacent nucleosomes parallel to each other, yielding an intrinsic rotational phase angle $\Psi_0 = 0$ between nucleosomes. For $\phi_0 < 0$, the twisting energy is minimized when $\alpha_i + \gamma_i = \phi_0$ (< 0). This leads to every nucleosome k oriented anticlockwise with respect to its earlier nucleosome $k-7$, i.e., $\Psi_0 < 0$. By the same argument, $\phi_0 > 0$ leads to positive phase angles, i.e., $\Psi_0 > 0$. Thus, altering ϕ_0, which is equivalent to changing DNA pitch, provides a convenient way of modulating the rotational phasing of nucleosomes without altering the length of each linker DNA. In this study, we considered ϕ_0 values ranging within -1 rad to $+0.5$ rad, which corresponds to Ψ_0 values in the range $-400°$ to $+200°$, as $\Psi_0 \equiv 7\phi_0$. To ensure that no twisting is introduced on the nucleosome-wrapped DNA, we set $\alpha^+_i + \gamma^+_i = \phi_0$.

The total electrostatic energy U_{el} is given by the total Debye-Hückel interactions between all linker DNA and nucleosomes beads except those directly connected to each other or part of the same rigid body (i.e., nucleosome)

$$U_{\mathrm{el}} = \sum_{i,j} \frac{q_i q_j}{4\pi\epsilon\epsilon_0 r_{ij}} \exp(-\kappa r_{ij}), \tag{S4}$$

where the sum runs over only interacting pairs of linker DNA or nucleosome beads, q_i and q_j are the charges on the interacting beads i and j, r_{ij} is the distance between the beads, κ is the inverse Debye length, ϵ is

the dielectric constant of the solvent, and ϵ_0 is the permittivity of free space. At 10 mM monovalent salt concentration, the nucleosome beads and linker DNA beads carry charges of $q_c = -6.02e$ and $q_l = -7.53e$, respectively, according to the parameterization procedure described in Ref. [5].

The total excluded volume energy U_{ev} is given by Lennard-Jones interactions between the same pairs of linker DNA or nucleosome beads used for computing the electrostatic energy

$$U_{\text{ev}} = k_{ev} \sum_{i,j} \left[\left(\frac{\sigma_{ij}}{r_{ij}} \right)^{12} - \left(\frac{\sigma_{ij}}{r_{ij}} \right)^{6} \right], \tag{S5}$$

where k_{ev} is an energy parameter and σ_{ij} is the effective size of the interacting pairs of beads i and j. $\sigma_{ij} = \sigma_{cc}$ when both i and j represent nucleosome beads, $\sigma_{ij} = \sigma_{ll}$ when both i and j represent linker DNA beads, and $\sigma_{ij} = \sigma_{cl}$ when i and j represent a linker DNA and nucleosome bead, or vice versa. The parameter values used to describe all the above potential energy functions are listed in Table S1.

2 Brownian dynamics simulations

The dynamics of the nucleosome arrays are computed by using Brownian dynamics (BD) simulations. A second-order Runge-Kutta algorithm [6] is employed to update the positions and orientations of all nucleosomes and linker DNA beads.

The translational equations of motion for component i is given by

$$\mathbf{r}_i(t + \Delta t) = \mathbf{r}_i(t) + \sum_{j=1}^{N} \frac{\mathbf{D}_{ij}(t) \cdot (\mathbf{F}_j(t) + \mathbf{F}_j^*)}{2k_{\text{B}}T} \Delta t + \Delta \mathbf{R}_i, \tag{S6}$$

where Δt is the time step, $\mathbf{F}_j(t)$ is the net force acting on the jth bead at time t, $\mathbf{F}_j^*$ is the predicted force at time $t + \Delta t$ as calculated below, $\mathbf{D}_{ij}$ is the hydrodynamic diffusion tensor, and $\Delta \mathbf{R}_i$ is a Gaussian-distributed random vector exhibiting the following mean and variance:

$$\langle \Delta \mathbf{R}_i \rangle = 0, \quad \langle \Delta \mathbf{R}_i \Delta \mathbf{R}_j \rangle = 2\mathbf{D}_{ij}(t) \Delta t \delta_{ij}, \tag{S7}$$

where δ_{ij} is the Kronecker delta function. Under low salt conditions, the arrays remain largely unfolded due to strong electrostatic repulsion between the linker DNA beads electrostatic interactions. This implies that the nucleosomes and linker DNA beads remain well separated and hydrodynamic interactions between them remain weak. In the absence of hydrodynamic interactions, the diffusion tensor is a diagonal matrix given by $\mathbf{D}_{ij} = (k_{\text{B}}T/6\pi\eta\sigma_i)\mathbf{I}$, where σ_i and η are the ith bead size and solvent viscosity, respectively.

The rotational equations of motion for component i is given by

$$\Omega_{\mathbf{x}_i}(t + \Delta t) = \Omega_{\mathbf{x}_i}(t) + \frac{D_{\mathbf{x}_i} \cdot (\tau_{\mathbf{x}_i}(t) + \tau_{\mathbf{x}_i}^*)}{2k_{\text{B}}T} \Delta t + \Delta W_i, \tag{S8}$$

where $\Omega_{\mathbf{x}_i}(t)$ represents the rotational state of the ith bead about its original coordinate system $\mathbf{x}_i \equiv \{\mathbf{a}_i, \mathbf{b}_i, \mathbf{c}_i\}$, $\tau_{\mathbf{x}_i}(t)$ is the torque on component i along $\mathbf{x}_i$ at time t, $D_{\mathbf{x}_i}$ is the rotational diffusion matrix, and ΔW_i is a Gaussian-distributed random number exhibiting the following mean and variance

$$\langle \Delta W_i \rangle = 0, \quad \langle \Delta W_i \Delta W_j \rangle = 2\Delta t D_{\mathbf{x}_i} \delta_{ij}. \tag{S9}$$

For linker DNA beads, only rotations about the $\mathbf{a}_i$ axis are allowed and the rotational diffusion constant is given by $D_{\mathbf{a}_i} = k_{\text{B}}T/4\pi\eta l_0 R_l^2$, where R_l is the hydrodynamic radius of linker DNA bead. For nucleosomes, rotations about all three axes are allowed and the rotational diffusion coefficient is given by $D_{\mathbf{a}_i} = D_{\mathbf{b}_i} = D_{\mathbf{c}_i} = k_{\text{B}}T/8\pi\eta R_c^3$, where R_c is the effective hydrodynamic radius of the nucleosome.

The net force acting on each component i is calculated from the gradient of the total potential energy U:

$$\mathbf{F}_j(t) = -\nabla_{\mathbf{r}_j} U. \tag{S10}$$

The net torque on each linker DNA bead acts only in the longitudinal direction $\mathbf{a}_i$ and is calculated as

$$\tau_{\mathbf{a}_i}(t) = -\frac{C}{l_0}(\alpha_i + \gamma_i - \alpha_{i-1} - \gamma_{i-1}). \tag{S11}$$

The net torque $\tau_{\mathbf{x}_i}(t)$ on each nucleosome i acts along all three coordinate axes and is given by

$$\tau_{\mathbf{x}_i}(t) = \tau_{F_i} + \tau_{B_i} + \tau_{T_i}, \tag{S12}$$

where τ_{F_i} is the torque acting on the nucleosome due to forces experienced by each nucleosome bead and τ_{B_i} and τ_{T_i} are associated with the bending and twisting of linker DNAs entering or exiting the nucleosome, respectively. Ref. [5] provides more detailed expressions of the three contributions.

In Eqs. S6 and S8, $\tau^*_{\mathbf{x}_i}$ and $\mathbf{F}^*_j$ are calculated at estimated positions $\mathbf{r}^*_i$ and orientations $\Omega^*_{\mathbf{x}_i}$ of nucleosomes and linker DNA beads at time $t + \Delta t$ obtained via

$$\mathbf{r}^*_i = \mathbf{r}_i(t) + \sum_{j=1}^{N} \frac{\mathbf{D}_{ij}(t) \cdot \mathbf{F}_j(t)}{k_{\mathrm{B}}T}\Delta t + \Delta \mathbf{R}_i, \tag{S13}$$

$$\Omega^*_{\mathbf{x}_i} = \Omega_{\mathbf{x}_i}(t) + \frac{D_{\mathbf{x}_i} \cdot \tau_{\mathbf{x}_i}(t)}{k_{\mathrm{B}}T}\Delta t + \Delta W_i. \tag{S14}$$

The time step, solvent viscosity, and hydrodynamic radii employed in our BD simulations are provided in Table S1.

3 Twisting protocol

We twist the nucleosome arrays by fixing the position and orientation of the first linker DNA bead, such that its frame $\{\mathbf{a}_1, \mathbf{b}_1, \mathbf{c}_1\}$ is oriented along the $\{\mathbf{z}, \mathbf{x}, \mathbf{y}\}$ axes, and rotating the last linker DNA frame $\{\mathbf{a}_N, \mathbf{b}_N, \mathbf{c}_N\}$ in a step-wise manner about $\mathbf{a}_N$ in $\Delta\Omega = \pm 45°$ increments. A stretching force of 0.34 pN, comparable with that used in the twisting experiments [2, 3], is also applied to the last linker DNA bead in the direction of the z-axis. This force is large enough to keep the arrays somewhat extended and small enough to keep the arrays in the entropic stretching regime where they remain conformationally dynamic.

The end linker DNAs are extended by attaching additional buffer DNA of length 21 nm ($\equiv$ 7 linker DNA beads) at both ends, which helps to minimize "boundary" effects on the terminal nucleosomes. However, the resulting end linker DNAs become too long and can easily become supercoiled, especially at the free end, by a small amount of external twisting, which would alter the extension-rotation behavior. To prevent this, the last 7 beads of the two end linker DNAs are trapped by a "confining" harmonic potential

$$U_{\mathrm{conf}} = \frac{K}{2}\left[\sum_{i=1}^{7}(x_i^2 + y_i^2) + \sum_{i=N-6}^{N}(x_i^2 + y_i^2)\right], \tag{S15}$$

where $K = 3.0$ kcal/mol/nm^2, and x_i and y_i represent the (x, y) coordinates of the ith linker DNA bead. This potential suppresses their supercoiling at large imposed rotations. The resulting confinement of the terminal linker DNA beads towards the $(0, 0, z)$ line also allows us to easily trace the array's extension along the z-axis. To conserve the array's topology during twisting, we introduce a repulsive "line" potential. The line potential is implemented by placing a set of rigidly held virtual linker DNA beads along two lines

extending in the $\pm z$ directions away from the two end linker DNA. These virtual beads interact with the linker DNA and nucleosome beads in the array through the Lennard-Jones potential with the same parameters used in Eq. S5:

$$U_{\text{line}} = k_{ev} \sum_{i,j} \left[\left(\frac{\sigma_{ij}}{r_{ij}} \right)^{12} - \left(\frac{\sigma_{ij}}{r_{ij}} \right)^{6} \right] \tag{S16}$$

where the sum i runs over the set of virtual beads and the sum j runs over all nucleosome and linker DNA beads. The purpose of these virtual beads is to prevent an array component from crossing over the array ends, thereby preserving the topology of the array.

To ensure that the measured extension-rotation curves are not biased by the initial topological state of the array at the onset of twisting, we performed 50 sets of twisting simulations at each value of Ψ_0, each simulation starting from a different equilibrated configuration of the array. Figure S2 shows the distribution in the orientation Ω_0 of the last linker DNA bead frame at the onset of twisting for arrays with $\Psi_0 = 80°$, where Ω_0 is the azimuth angle of the binormal vector projected onto a stationary reference frame $\Omega_0 = \text{acos}(\mathbf{b}_N \cdot \mathbf{x})$. Though individual arrays exhibit different starting orientations, the mean (15.7°) and standard deviation (89.5°) in Ω_0 are clearly negligible compared to the amount of rotations ($n > 10 \equiv 3600°$) imposed on the end linker DNA.

4 Topological properties of the arrays

4.1 Calculation of Linking number

We monitor topological changes in the nucleosome arrays during twisting by computing the linking number Lk, which is calculated as the sum of twist and writhe:

$$Lk = Tw + Wr. \tag{S17}$$

Since the nucleosome-wrapped DNA cannot store any twist in our model, the DNA twist Tw is calculated from the twist in the linker DNAs alone:

$$Tw = \frac{1}{2\pi} \sum_i \phi_i, \tag{S18}$$

where $\phi_i = \alpha_i + \gamma_i$ represents the twist angle between adjacent linker DNA frames.

To calculate DNA writhe Wr, we require the 3D path of the DNA axis running through the array. This is achieved by placing virtual beads along the expected helical trajectory of wrapped DNA within each nucleosome connecting the linker DNA entry and exit sites. The DNA writhe calculation also requires a closed path. This is achieved by introducing additional virtual beads to the two ends of the arrays and placing them in the configuration shown in Fig. S3a to generate a closed loop [7, 8]. We collectively denote the coordinates of the linker DNA beads and the virtual beads describing both the wrapped DNA trajectories and loop closure by $\mathbf{r}_i^{\text{d}}$ ($i = 1, \cdots, N_{\text{d}}$), where N_{d} is the total number of beads describing the DNA loop. The writhe can then calculated by using the discretized formula [9]:

$$Wr = \frac{1}{4\pi} \sum_{j=1}^{N_{\text{d}}} \sum_{i \neq j}^{N_{\text{d}}} \frac{\left[\left(\mathbf{r}_{j+1}^{\text{d}} - \mathbf{r}_j^{\text{d}} \right) \times \left(\mathbf{r}_{i+1}^{\text{d}} - \mathbf{r}_i^{\text{d}} \right) \right] \cdot \left(\mathbf{r}_j^{\text{d}} - \mathbf{r}_i^{\text{d}} \right)}{\left| \mathbf{r}_j^{\text{d}} - \mathbf{r}_i^{\text{d}} \right|^3}. \tag{S19}$$

The computed Lk for untwisted arrays at different Ψ_0 values are listed in Table S2. The relative contributions of Tw and Wr towards Lk vary with Ψ_0. Specifically, Wr remains negative for all Ψ_0 due to the left-handed wrapping of DNA in nucleosomes, while Tw changes its sign with Ψ_0 due to the twisting of linker DNA as it is under- and over-twisted by $\Psi_0 < 0$ and $\Psi_0 > 0$, respectively.

4.2 Determination of array's handedness

To assess global supercoiling of the arrays, we computed the writhe of the array "skeleton" that excludes the contribution of writhe from the wrapping of DNA within nucleosomes. To this end, we considered a different closed path connecting the centers of adjacent nucleosomes with straight lines and the same set of virtual beads used earlier for closing the loop. The coordinates of N_s such beads making up this path are collectively denoted by $\mathbf{r}_i^\mathrm{s}$ $(i = 1, \cdots, N_\mathrm{s})$ and the writhe is computed as before:

$$Wr_\mathrm{s} = \frac{1}{4\pi} \sum_{j=1}^{N_\mathrm{s}} \sum_{i \neq j}^{N_\mathrm{s}} \frac{[(\mathbf{r}_{j+1}^\mathrm{s} - \mathbf{r}_j^\mathrm{s}) \times (\mathbf{r}_{i+1}^\mathrm{s} - \mathbf{r}_i^\mathrm{s})] \cdot (\mathbf{r}_j^\mathrm{s} - \mathbf{r}_i^\mathrm{s})}{|\mathbf{r}_j^\mathrm{s} - \mathbf{r}_i^\mathrm{s}|^3}. \tag{S20}$$

The array's handedness is then determined as $Wr_\mathrm{s} < 0$ and $Wr_\mathrm{s} > 0$ for left- and right-handed arrays, respectively.

4.3 Determination of nucleosomal states

To determine whether a nucleosome exhibits an open, negative, or positive state, we used entry and exit vectors $\mathbf{l}_i^\mathrm{en}$ and $\mathbf{l}_i^\mathrm{ex}$ depicted in Fig. S1c, and as defined by

$$\begin{aligned} \mathbf{l}_i^\mathrm{en} &= \mathbf{r}_i^- - \mathbf{r}_{i-7}^+, \\ \mathbf{l}_i^\mathrm{ex} &= \mathbf{r}_{i+7}^- - \mathbf{r}_i^+, \end{aligned} \tag{S21}$$

where $\mathbf{r}_{i-7}^+$ and $\mathbf{r}_{i+7}^-$ are the linker DNA exit and entry points for the two neighboring nucleosomes, respectively. We then consider two planes defined by $(\mathbf{l}_i^\mathrm{en}, \mathbf{c}_i^-)$ and $(\mathbf{l}_i^\mathrm{ex}, \mathbf{c}_i^\mathrm{DNA})$, whose normal vectors are given by:

$$\begin{aligned} \mathbf{v}_1 &= \mathbf{l}_i^\mathrm{en} \times \mathbf{c}_i^-, \\ \mathbf{v}_2 &= \mathbf{l}_i^\mathrm{ex} \times \mathbf{c}_i^\mathrm{DNA}. \end{aligned} \tag{S22}$$

We next compute the vector $\mathbf{v}_3 = \mathbf{v}_2 \times \mathbf{v}_1$, which lies parallel to the above two planes and is also either parallel or anti-parallel to the normal of nucleosome $\mathbf{c}_i$. Then, one can determine whether the ith nucleosome is in the open or crossed state as follows:

$$\mathbf{c}_i \cdot \mathbf{v}_3 = \begin{cases} < 0, & \text{open state} \\ > 0, & \text{crossed state.} \end{cases}$$

Once a nucleosome has been declared as a crossed state, we next determine whether it is negatively or positively crossed. To this end, we construct a small loop connected with following position vectors (Fig. S1c):

$$\mathbf{r}_\mathrm{nucl}^\mathrm{loop} = \{\mathbf{r}_{i-7}^+ \to \mathbf{r}_i^- \to \mathbf{r}_i \to \mathbf{r}_i^+ \to \mathbf{r}_{i+7}^- \to \mathbf{r}_{i-7}^+\}. \tag{S23}$$

We then calculate the writhe Wr_nucl over this closed loop using Eq. S20, which allows us to distinguish two opposite crossings between the entry and exit linkers, as $Wr_\mathrm{nucl} < 0$ and $Wr_\mathrm{nucl} > 0$ for negatively and positively crossd state, respectively.

5 Torsional rigidity and torque calculation

To examine the torsional resilience of our arrays, we obtained the total potential energy U of the arrays as functions of imposed rotations n. Fig. 6a and b shows the two profiles for arrays with different Ψ_0 (*main text*). Fitting the total energy profile to the function $U = \frac{C_\mathrm{array}}{2l_0}(2\pi n - 2\pi n_0)^2$ allows us to extract the

effective torsional rigidity C_{array} of the arrays. We obtain $C_{\text{array}} \approx 0.30$ kcal/mol nm for $\Psi_0 = -80^\circ$ (inset, Fig. 6a), which is much smaller than that measured for naked DNA $C_{\text{DNA}} = 57$ kcal/mol nm.

The total energy profiles also allows us to estimate the torque (Fig. 6b). In our simulation scheme, the torque is generated by rotating the last linker DNA frame, which generally requires an energy of $\tau\Delta\Omega$, where τ is a magnitude of the torque. Assuming that the time interval $\Delta t_{\text{eq}} = 2\,\mu$s between rotations of the end frame is sufficiently large to equilibrate the arrays, the torque can be obtained via $U \approx \tau\Omega = \tau(2\pi n)$.

References

[1] Davey CA, *et al.*, *Solvent mediated interactions in the structure of the nucleosome core particle at 1.9 Å resolution*, J Mol Biol, 319, (2002), pp. 1097–1113.

[2] Bancaud A, *et al.*, *Structural plasticity of single chromatin fibers revealed by torsional manipulation*, Nat Struct Mol Biol, 13, (2006), pp. 444–450.

[3] Celedon A, *et al.*, *Magnetic Tweezers Measurement of Single Molecule Torque*, Nano Letters, 9, (2009), pp. 1720–1725.

[4] Arya G and Schlick T, *A tale of tails: how histone tails mediate chromatin compaction in different salt and linker histone environments*, J Phys Chem A, 113, (2009), pp. 4045–4059.

[5] Beard DA and Schlick T, *Computational modeling predicts the structure and dynamics of chromatin fiber*, Structure, 9, (2001), pp. 105–114.

[6] Iniesta A and de la Torre JG, *A second-order algorithm for the simulation of the Brownian dynamics of macromolecular models*, J Chem Phys, 92 (1990), pp. 2015–2018.

[7] Vologodskii AV and Marko JF, *Extension of Torsionally Stressed DNA by External Force*, Biophys J, 73, (1997), pp. 123–132.

[8] Rossetto V and Maggs AC, *Writhing geometry of open DNA*, J Chem Phys, 21, (2005), pp. 9864–9874.

[9] Chirico G and Langowski J, *Kinetics of DNA supercoiling studied by Brownian Dynamics Simulation*, Biopolymers, 34, (1994), pp. 415–433.

[10] Horowitz DS and Wang JC, *Torsional rigidity of DNA and length dependence of the free energy of DNA supercoiling*, J Mol Biol, 173, (1984), pp. 75–91.

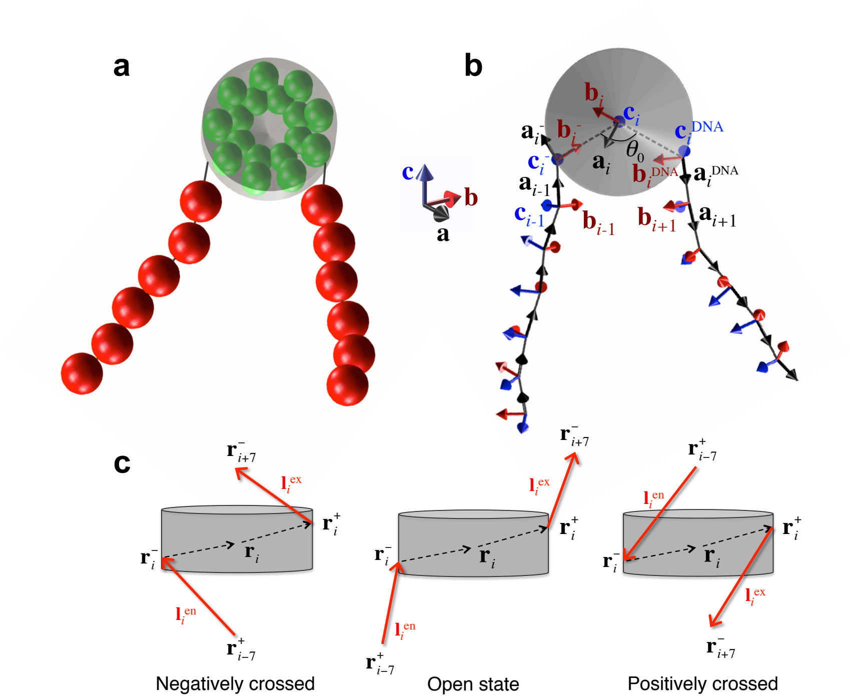

Figure S1: (a) Schematic representation of the coarse-grained model of nucleosome array. Each linker DNA is represented by 6 beads (red) and each nucleosome by 26 beads (green). (b) Model geometry showing the different position vectors and orientation frames assigned to linker DNA beads and the nucleosome. (c) Schematic of vectors used for differentiating between open, negative, and positive nucleosome states .

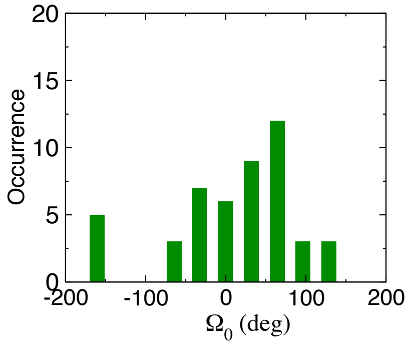

Figure S2: Angular distribution of the end linker DNA bead frame of the initial equilibrated arrays with $\Psi_0 = 80°$

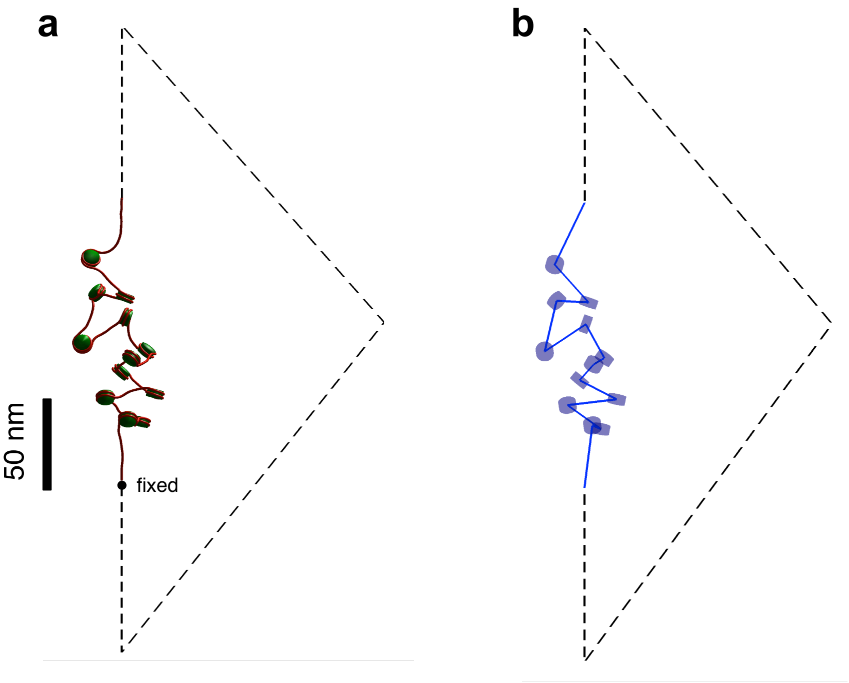

Figure S3: DNA axis (a) and array skeleton (b) paths used for computing DNA writhe Wr and global writhe Wr_s. The dotted lines in (a) and (b) represent the path of the virtual beads used for closing the arrays and the blue lines in (b) represent straight lines between the centers of nucleosomes used in calculating Wr_s.

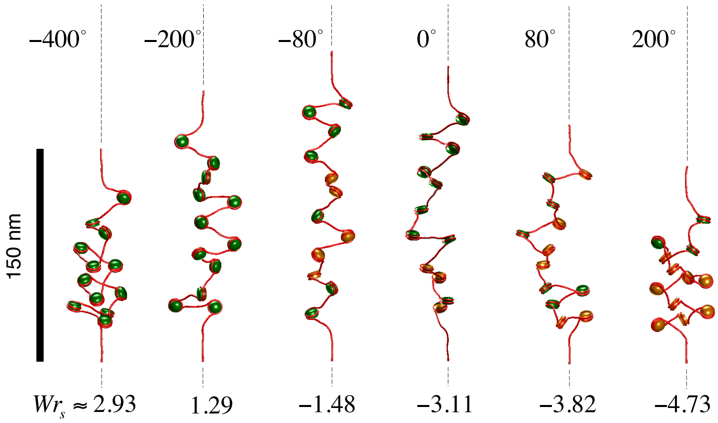

Figure S4: Representative snapshots of tension-equilibrated arrays with different Ψ_0 showing how the array extension and handedness changes with nucleosome phasing. From left to right, the arrays transform from right- to left-handed coils, as indicated by Wr_s changing from positive to negative values, respectively.

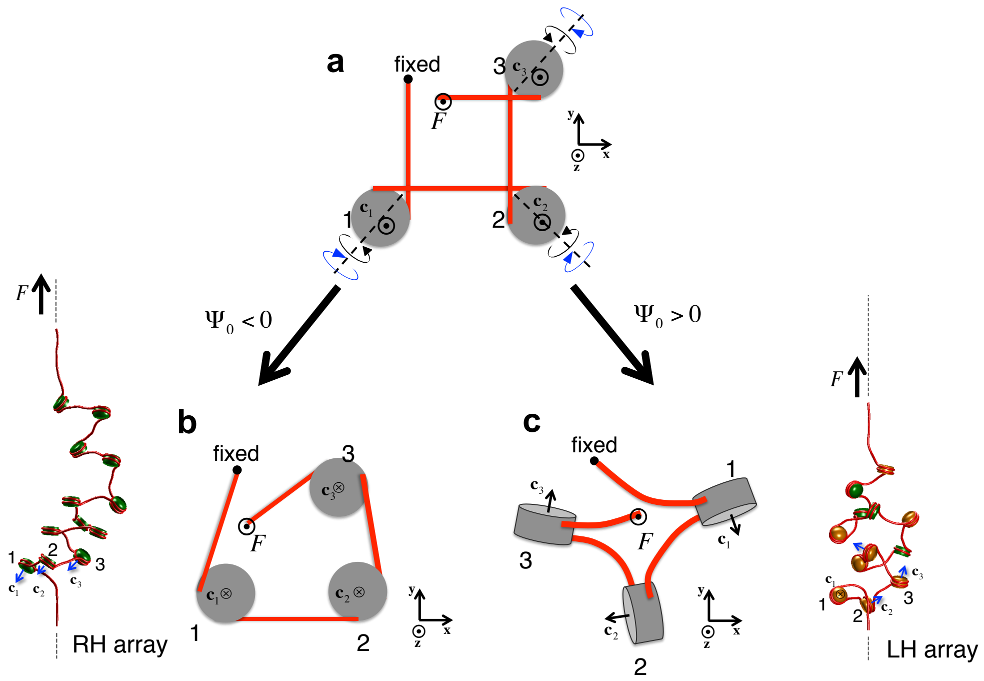

Figure S5: Schematic explanation for arrays forming right-handed (RH) and left-handed (LH) coils for $\Psi_0 < 0$ and $\Psi_0 > 0$, respectively. (a) The array with $\Psi_0 = 0$ is under a stretching force along the $+z-$direction, causing nucleosomes to undergo clockwise rotation (rounded arrow in black), leading to their open state. (b) For $\Psi_0 < 0$, the linker DNA imposes an additional rotation to the nucleosome in the same direction as that by the force, which further stabilizes the open state, leading to stable RH coils. For extreme Ψ_0, all nucleosomes flip completely over to -z-direction, as shown in a representative configuration obtained from the simulation. (c) For $\Psi_0 > 0$, the linker DNA imposes anticlockwise rotation to the nucleosomes (rounded arrow in blue), which turns them into the negative state and rearranges the nucleosomal trajectory into LH coils. In this case, the normal vectors lie along the helical trajectory of the array.

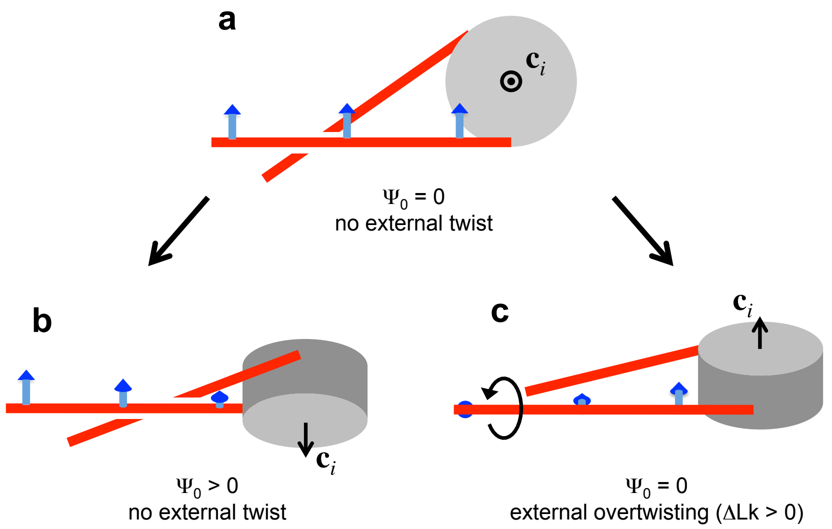

Figure S6: Schematic explaining the differential response of a nucleosome to intrinsic overtwisting of DNA ($\Psi_0 > 0$) versus external overtwisting ($\Delta Lk > 0$). (a) Equilibrium conformation of the nucleosome at $\Psi_0 = 0$. (b) When $\Psi_0 > 0$, the linker DNA induces anticlockwise rotation of nucleosome, leading to crossed linker DNAs. (c) When $\Delta Lk > 0$, it induces the clockwise rotation of the nucleosome, leading to open linker DNAs.

Table S1: Parameter values employed in BD simulations of nucleosome arrays

N_c	Total number of nucleosomes in the array	12
N	Total number of linker and core beads in the array	104
A	Stretching constant of DNA	55 $k_B T/$ nm^2
B	Bending constant of DNA	$(L_p/l_0)k_B T$
C	Torsional constant of DNA	97 $k_B T$ nm
l_p	Persistence length of DNA	50 nm
θ_0	Separation angle between entry and exit linkers at core	120°
l_0	Equilibrium bond length of linker DNAs	3 nm
$2w_0$	Width of the nucleosomal DNA	3.6 nm
r_0	Radius of the nucleosomal DNA	4.8 nm
R_c	Hydrodynamic radius of nucleosome core	5.46 nm
R_l	Hydrodynamic radius of linker bead	1.5 nm
η	Water viscosity	10^{-3} Pa · s
σ_{cc}	Core/core excluded volume diameter	2.0 nm
σ_{cl}	Core/linker excluded volume diameter	3.0 nm
σ_{ll}	Linker/linker excluded volume diameter	3.6 nm
k_{ev}	Excluded volume energy parameter	1 $k_B T$
q_l	Charge on a linker DNA bead	$-7.53e$
q_c	Charge on a core disk bead	$-6.02e$
κ	Inverse Debye screening length	0.33 nm^{-1}
ϵ	Dielectric constant of solvent	80
Δt	BD simulation timestep	2 ps

Table S2: Partitioning of linking number into twist and writhe

Ψ_0	$\langle Wr \rangle$	$\langle Tw \rangle$	$\langle Lk \rangle$	$(\langle Wr \rangle - \langle Wr_s \rangle)/N_C$*
−400°	−9.88	−15.67	−25.58	−1.07
−200°	−8.66	−7.95	−16.19	−0.83
−80°	−12.14	−3.28	−15.42	−0.89
0°	−14.17	−0.22	−14.39	−0.92
80°	−15.12	2.98	−12.13	−0.94
200°	−16.55	7.46	−9.09	−0.98

*This gives the writhe of nucleosomal DNAs.
