# Supplementary material for: Torsional behavior of chromatin is modulated by rotational phasing of nucleosomes
Source: Nucleic Acids Res. 2014 Aug 6;42(15):9691–9. doi: 10.1093/nar/gku694 (PMC4150795; doi:10.1093/nar/gku694)
Supplement: SUPPLEMENTARY DATA [file supp_gku694_nar-01019-m-2014-File009.doc]

**MOVIE LEGENDS**

Conformational changes of 12-nucleosome arrays for all tested Ψ_0_ under external twisting. The numbers at the bottom of each movie indicate the number of imposed turns *n*, *n*$< 0$and *n* $> 0$for negative and positive rotations, respectively.

Video S1 for Ψ_0_ = −400^o^

Video S2 for Ψ_0_ = −200^o^

Video S3 for Ψ_0_ = −80^o^

Video S4 for Ψ_0_ = 0^o^

Video S5 for Ψ_0_ = 80^o^

Video S6 for Ψ_0_ = 200^o^
